# Transcriptional responses of *Biomphalaria pfeifferi* and *Schistosoma mansoni* following exposure to niclosamide, with evidence for a synergistic effect on snails following exposure to both stressors

**DOI:** 10.1371/journal.pntd.0006927

**Published:** 2019-12-16

**Authors:** Sarah K. Buddenborg, Bishoy Kamel, Lijing Bu, Si-Ming Zhang, Gerald M. Mkoji, Eric S. Loker

**Affiliations:** 1 Department of Biology, Center for Evolutionary and Theoretical Immunology, University of New Mexico, Albuquerque NM United States of America; 2 Wellcome Sanger Institute, Wellcome Genome Campus, Hinxton United Kingdom; 3 Center for Biotechnology Research and Development, Kenya Medical Research Institute, Nairobi KEN; Case Western Reserve University, UNITED STATES

## Abstract

**Background:**

Schistosomiasis is one of the world’s most common NTDs. Successful control operations often target snail vectors with the molluscicide niclosamide. Little is known about how niclosamide affects snails, including for *Biomphalaria pfeifferi*, the most important vector for *Schistosoma mansoni* in Africa. We used Illumina technology to explore how field-derived *B*. *pfeifferi*, either uninfected or harboring cercariae–producing *S*. *mansoni* sporocysts, respond to a sublethal treatment of niclosamide. This study afforded the opportunity to determine if snails respond differently to biotic or abiotic stressors, and if they reserve unique responses for when presented with both stressors in combination. We also examined how sporocysts respond when their snail host is treated with niclosamide.

**Principal findings:**

Cercariae-producing sporocysts within snails treated with niclosamide express ~68% of the genes in the *S*. *mansoni* genome, as compared to 66% expressed by intramolluscan stages of *S*. *mansoni* in snails not treated with niclosamide. Niclosamide does not disable sporocysts nor does it seem to provoke from them distinctive responses associated with detoxifying a xenobiotic. For uninfected *B*. *pfeifferi*, niclosamide treatment alone increases expression of several features not up-regulated in infected snails including particular cytochrome p450s and heat shock proteins, glutathione-S-transferases, antimicrobial factors like LBP/BPI and protease inhibitors, and also provokes strong down regulation of proteases. Exposure of infected snails to niclosamide resulted in numerous up-regulated responses associated with apoptosis along with down-regulated ribosomal and defense functions, indicative of a distinctive, compromised state not achieved with either stimulus alone.

**Conclusions/Significance:**

This study helps define the transcriptomic responses of an important and under-studied schistosome vector to *S*. *mansoni* sporocysts, to niclosamide, and to both in combination. It suggests the response of *S*. *mansoni* sporocysts to niclosamide is minimal and not reflective of a distinct repertoire of genes to handle xenobiotics while in the snail host. It also offers new insights for how niclosamide affects snails.

## Author summary

Schistosomaisis control programs often employ the use of chemical molluscicides, such as niclosamide, to control the obligatory intermediate snail hosts. Despite its widespread use, we know little about how niclosamide affects snails like *Biomphalaria pfeifferi*, the most important vector *Schistosoma mansoni* in Africa. Following sequencing the transcriptomes of uninfected and *S*. *mansoni-*infected *B*. *pfeifferi* treated with niclosamide, we analyze the snail’s response to both biotic and abiotic stressors. We can also examine the response of *S*. *mansoni* to niclosamide exposure during intramolluscan development. *Biomphalaria pfeifferi* snails exposed only to niclosamide showed unique up-regulation of stress and defense-related transcripts not seen in snails infected with a biotic stressor like *S*. *mansoni* infection. *Schistosoma mansoni*-infected *B*. *pfeifferi* treated with niclosamide were clearly unable to regulate normal metabolic and detoxification processes. Cercariae-producing sporocysts within snails treated with niclosamide are largely unaffected and continue to produce transcripts required for cercariae production.

## Introduction

Schistosomiasis control remains elusive in many of the world’s hyperendemic foci of infection in sub-Saharan Africa, jeopardizing the goals of diminishing schistosomiasis as a public health concern, or of eliminating transmission where possible by 2025 [1]. Several recent papers have called for the need to adopt more integrated control approaches instead of relying on chemotherapy alone to achieve eventual elimination [2–3], and there has been a resurgence in interest in methods to control the snails that vector human schistosomiasis [4–5]. Although the practical options available for use in snail control remain limited, molluscicides have been advocated because there are several recorded instances where their use has been associated with successful control [4,6].

Following the discovery of niclosamide’s molluscicidal properties in the 1950s, it has been incorporated into the commercial preparation known as Bayluscide [7] and is the only molluscicide approved for use in schistosomiasis control by the WHO Pesticide Evaluation Scheme (WHOPES). Use of niclosamide has enjoyed a modest resurgence and its focal application in snail control is advocated by WHO [8]. It has been used widely in Egypt and China as a mainstay for control operations, and it is used in both experimental [9–10] and in new control contexts, most notably recently as part of the *S*. *haematobium* elimination program in Zanzibar [11–12].

Although some work on the effects of molluscicides on oxygen consumption and carbohydrate metabolism of snails has been undertaken [13–14], there have been relatively few studies employing modern techniques to assess the impacts of molluscicide exposure on schistosome-transmitting snails. Zhao et al. [15], working with the amphibious snail *Oncomelania hupensis*, the intermediate host for *Schistosoma japonicum*, undertook an Illumina-based *de novo* transcriptome study to show this snail responded to two novel niclosamide-based molluscicides by up-regulating production of two cytochrome p450 (CYPs) genes, and one glutathione-S-transferase. Zhang et al. [16] examined the effects of three different sublethal concentrations of niclosamide (0.05, 0.10, and 0.15 mg/L for 24 hours) on the transcriptional activity of *Biomphalaria glabrata* as examined using an oligonucleotide microarray and noted up-regulation of several genes associated with biotransformation of xenobiotics (CYPs and glutathione-S-transferase), drug transporters, heat shock proteins (HSP 20, 40 and 70 families) and vesicle trafficking. Down-regulated hemoglobin production was also noted. Niclosamide is able to kill schistosome miracidia and cercariae [17–18] and field experiments in China have shown that niclosamide is effective at reducing the number of viable *S*. *japonicum* cercariae in streams and downstream infection of sentinel mice [19].

With respect to the effects of niclosamide on schistosome-infected snails, or on the schistosome sporocysts within them, there has been remarkably little study. Sturrock [20] investigated the effects of sublethal concentrations of niclosamide on infections of *S*. *mansoni* on *Biomphalaria sudanica tanganyicensis* and noted that: 1) snails treated with molluscicide that survived were still susceptible to infection; 2) snails with prepatent infections were not initially more susceptible to molluscicide but had slightly delayed rate of parasite development and production of cercariae and did eventually exhibit higher mortality as they entered patency; and 3) survivorship of snails exposed during the patent period was less, although it takes some time for the effect to occur. Sturrock [20] commented that the combined stress of producing cercariae and exposure to molluscicide likely contributed to the higher mortality rate in patent snails. He also noted that doses sufficiently high to kill schistosome sporocysts in snails were probably above the lethal doses needed to kill the snails themselves.

In this study, building on the microarray results of Zhang et al. [16] with *B*. *glabrata*, we sought to obtain a more in-depth view of the transcriptome of molluscicide-exposed snails by using the Illumina platform to examine the responses of *Biomphalaria pfeifferi* to a sublethal dose (0.15 mg/L) of niclosamide. *Biomphalaria pfeifferi* is widely distributed in streams, ponds and impoundments in Africa and is probably responsible for transmitting more cases of *Schistosoma mansoni* than any other *Biomphalaria* species [21–22]. In addition, we examined the transcriptional responses to the same dose of molluscicide of *B*. *pfeifferi* harboring cercariae-producing *S*. *mansoni* infections. We were able to compare the responses of the above snails to both uninfected and infected *B*. *pfeifferi* not treated with molluscicides (see companion studies [23,24]). For both the previous and present studies, we chose to examine the responses of snails recently removed from field habitats and therefore considered to be more representative of what might be expected of snails comprising natural populations actually treated with molluscicides. The approach taken enables us to ascertain if and how the transcriptional responses of snails already coping with a massive *S*. *mansoni* infection can be further altered by simultaneous exposure to a toxic xenobiotic. For example, might snail genes up-regulated following exposure to *S*. *mansoni* trend towards down-regulation if the snail is treated with niclosamide and required to produce increased quantities of molecules involved in detoxification?

With respect to the sporocysts of *S*. *mansoni* residing in snails treated with niclosamide, do they exhibit any tendency to express genes that are not normally expressed during intramolluscan development, and if so, do the ensuing proteins favor survival of the sporocysts or of the stressed snail in which the sporocysts reside? Three possible scenarios for *S*. *mansoni* transcriptional response to molluscicide exposure can be considered: 1) We see an overall absence of *S*. *mansoni* transcripts indicating suspension of activity; 2) Cercariae-producing *S*. *mansoni* sporocysts express unique features that are absent in response to molluscicide exposure; and 3) Shedding *S*. *mansoni* stages treated with molluscicide show unique transcriptional responses suggestive of a hitherto unseen ability to protect the host-parasite unit in which they reside from a xenobiotic.

## Methods

### Ethics statement

This project was undertaken with approval of Kenya’s National Commission for Science, Technology, and Innovation (permit number NACOSTI/P/15/9609/4270), National Environment Management Authority (NEMA/AGR/46/2014) and an export permit has been granted by the Kenya Wildlife Service (0004754).

*Biomphalaria pfeifferi* used in Illumina sequencing were collected from Kasabong stream in Asembo Village, Nyanza Province, western Kenya (34.42037°E, 0.15869°S) and transferred to our field lab at The Centre for Global Health Research (CGHR) at Kisian, western Kenya. Snails sized 6-9mm in shell diameter were placed under natural light to check for shedding of digenetic trematode cercariae [25]. Snails shedding only *S*. *mansoni* cercariae and uninfected, non-shedding snails were held in aquaria for one day. After cleaning shells with 70% EtOH, whole shedding and uninfected snails (the two control groups) were placed individually into 1.5ml tubes with 1ml of TRIzol (Invitrogen, Carlsbad CA) and stored at -80°C until extraction. Additional *B*. *pfeifferi* confirmed to be uninfected and *S*. *mansoni-*shedding (patent infections) snails were treated with a concentration of 0.15 mg/L niclosamide (Sigma-Aldrich, St. Louis MO) with final DMSO concentrations at 1/1000 (v/v) for 24 hours at 26-28°C with aeration [16]. Previous 24 hour exposure of *B*. *glabrata* to varying doses of niclosamide (0.05mg/L, 0.10mg/L, and 0.15mg/L) found that the 0.15mg/L dose produced the most robust transcriptional response, as assessed by microarray analysis [16]. All snails treated with 0.15mg/L niclosamide were alive and responding after the 24 hours dosage period. Therefore, a 0.15mg/L dose was also selected for this study as the sublethal dose administered to *B*. *pfeifferi*. Our previous paper using a microarray on *B*. *glabrata* treatment with niclosamide contained control snails with DMSO at 1/000 (v/v) and there was no noticeable effect on transcriptional levels attributable to DMSO. Also, control assays with DMSO showed no effects to *B*. *truncatus* snails, the intermediate host of *Schistosoma haematobium* [26]. For these reasons, we did not include an additional group of control snails treated to DMSO.

Three snails from each of the four sample groups were chosen as biological triplicates for Illumina Hi-Seq sequencing performed at the National Center for Genome Resources (NCGR) in Santa Fe, NM. RNA extraction, library preparation, and sequencing procedures can be found in Buddenborg et al. [23,24]. Illumina RNA sequencing reads underwent extensive processing in order to separate host, parasite, and potential symbiont reads. *Biomphalaria pfeifferi* read quantification and differential expression analyses for snail CDS (coding sequences; the coding region of a gene) were performed using RSEM (RNA-Seq by expectation maximization) [27] and EBSeq [28]. *Biomphalaria pfeifferi* with a posterior probability of differential expression (PPDE) > = 0.95 were considered significant. Read counts acquired from RSEM *S*. *mansoni* and TPM (Transcripts Per kilobase Million) values were used for downstream analyses. TPM is calculated by normalizing for transcript length and then by sequencing depth ultimately allowing us to compare the proportion of reads that mapped to a specific transcript [29]. The raw and assembled sequence data are available at NCBI under BioProject ID PRJNA383396. Raw read counts and normalized read counts can be found in S1 File and S2 File. In one snail, uninfected replicate 3 (Bp replicate 3) we recovered platyhelminth reads consistent with *Ribeiroia* (described at length in [23]). It is likely this snail had been exposed to *Ribeiroia*, and as such, we removed this sample for our current analysis as its effects on comparisons with *B*. *pfeifferi* treated with molluscicide could not be determined. MDS (multidimensional scaling) plots for each pairwise comparison performed in our analyses are provided in S1 Fig.

## Results and discussion

### Overall *B*. *pfeifferi* and *S*. *mansoni* transcriptomic responses to molluscicide exposure

**Fig 1:**
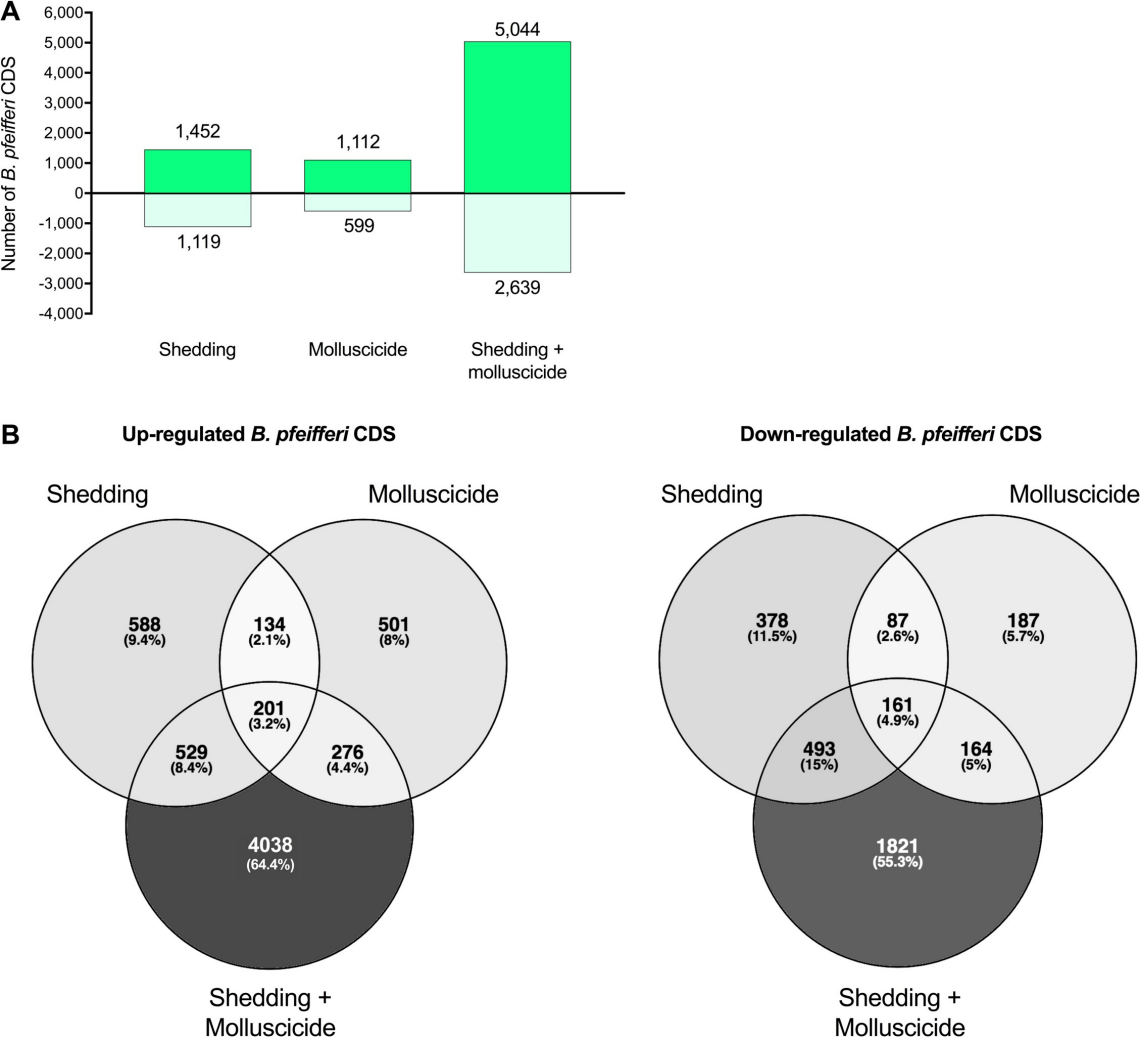
(A) Number of *B*. *pfeifferi* CDS (protein-coding sequences) up- and down-regulated in *S*. *mansoni-*infected snails untreated (Shedding), uninfected but molluscicide-treated snails (Molluscicide), and snails with both *S*. *mansoni*-infected and molluscicide treatment (Shedding + Molluscicide) when compared to uninfected untreated snails. (B) Venn diagrams showing shared and unique *B*. *pfeifferi* CDS between differentially expressed groups.

Relative to uninfected and untreated control *B*. *pfeifferi*, the overall differential gene expression responses were measured for snails i) with shedding *S*. *mansoni* infections only, ii) treated for 24 hours to a sublethal dose of niclosamide only, or iii) harboring shedding *S*. *mansoni* infections *and* treated to niclosamide (Fig 1A). The responses of shedding snails relative to untreated uninfected controls is discussed extensively by Buddenborg et al. [23] and the *S*. *mansoni* intramolluscan response is reported in Buddenborg et al [24]. With respect to molluscicide exposure, this is the first Illumina-based view of the transcriptomics response for any species of planorbid snail, and supplements and extends the view provided by the microarray study for uninfected *B*. *glabrata* of Zhang et al. [16]. Zhao et al. [15] undertook an Illumina-based study of the molluscicide-induced transcriptome of *Oncomelania hupensis*, the pomatiopsid snail host of *S*. *japonicum*. The response of *B*. *pfeifferi* to simultaneous exposure to schistosome infection and niclosamide treatment is the first glimpse we have for how snails respond transcriptionally to simultaneous exposure to these two relevant stressors.

For each of the three groups noted, the number of up-regulated snail features exceeded the number of down-regulated features. For both up- and down-regulated features, it was remarkable that over half of the transcripts proved to be distinctively represented in the combined *S*. *mansoni*-infected and molluscicide-exposed group (Fig 1B). Over 4,000 genes were distinctively up-regulated in the snails receiving the combination of stressors. This was the largest number found in any single group of either venn diagram. It was surprising to us that larger numbers of genes were not found in the cells of either venn diagram that represented two or all three of the groups. It was also evident that although the response of niclosamide-exposed snails had features in common to those evoked by *S*. *mansoni* exposure, many genes were also uniquely differentially expressed by exposure to just niclosamide. Further inspection of the pattern in expression levels exhibited by genes uniquely expressed in the combined *S*. *mansoni*-infected and molluscicide-treated group revealed that in comparison to genes represented in other cells, they were modest in the degree of their differential expression. The specific nature of the genes responsive to either molluscicide alone, or to molluscicides and *S*. *mansoni* are discussed further below.

The transcriptomic responses of intramolluscan stages of *S*. *mansoni*, including those from snails actively shedding cercariae are described by Buddenborg et al. [24], and are supplemented here by responses of shedding snails treated with niclosamide (S2 Fig). *Schistosoma mansoni* from shedding snails shared 80.6% of expressed transcripts with *S*. *mansoni* from shedding snails treated with sublethal niclosamide at similar expression levels. *Schistosoma mansoni* treated with niclosamide expressed 19% more transcripts, but this response was variable among replicates, and >90% of these extra transcripts were expressed less than 2 log_2_ normalized counts when replicate counts were averaged.

### Responses of *S*. *mansoni* cercariae-producing sporocysts within *B*. *pfeifferi* exposed to sublethal niclosamide treatment

Sturrock [20] noted that the lethal dose of niclosamide for intramolluscan schistosomes is higher than what is needed to kill the host snail. This may be the case because *S*. *mansoni* sporocysts are protected both by their own syncytial tegument [30] and by being embedded in the host snail’s tissues. At least with respect to a 24 hour treatment to a sublethal niclosamide dose for *B*. *pfeifferi*, we did not see extensive and broad down-regulation or absence of *S*. *mansoni* transcripts involved in the following processes that would indicate a direct affect to niclosamide: nutrient uptake across the tegument, cercariae production, or germ ball development and proliferation.

Defense and stress responses were sustained in niclosamide-treated sporocysts relative to untreated sporocysts. Peroxiredoxins like glutathione peroxidase and thioredoxin peroxidase, which may be responsible for elimination of potentially lethal hydrogen peroxide produced by the snail [31–33], were stably maintained, as were protective responses like planarian-like bacterial defense homologs, heat shock proteins, and SODs (S3 Fig). Particularly noteworthy is the lack of an obvious response of the single *S*. *mansoni* cytochrome p450 gene to niclosamide presence. As noted by Ziniel et al. [34] and by us previously [24], parasitic helminths in general lack extensive cytochrome p450 repertoires, quite unlike *B*. *pfeifferi* which does deploy cytochrome p450 responses upon exposure to molluscicides (see below). Drug efflux transporters like ABC transporters, known to be up-regulated in adult schistosomes expose to praziquantel [35–36], are expressed in cercariae-producing *S*. *mansoni* sporocysts [24] but do not show any conspicuous change in their expression pattern following exposure to niclosamide (S4 Fig). Our results agree with a study on the liver fluke *Fasciola gigantica* treated with rhodamine-labeled niclosamide that also lacked substantial changes in ABC transporter activity [37].

*S*. *mansoni* sporocysts, and the cercariae developing within them, express a diverse array of proteases, including elastases and leishmanolysins [24,38], with likely functions in disabling snail defenses, dissolution of snail tissues to provide living space, facilitating intra-snail migration of sporocysts and for packaging in cercariae which use them both for exiting the snail host and entering the mammalian definitive host. Protease inhibitors are also produced and likely counteract proteases that the snail expresses late in infection [24,39]. The overall patterns of expression of proteases or protease-inhibitors did not differ substantially between sporocysts in untreated and niclosamide-treated snails (S5 Fig).

The modest increases in proteases, transporters, germinal cell proliferation factors and neuropeptide or neural development markers [40–46] in niclosamide-exposed *in vivo* sporocysts (S6 Fig) all serve to further highlight the fact that the 24 h niclosamide exposure we used was certainly not lethal to the sporocysts nor did it seem to significantly curtail their transcriptional production or to invoke transcripts associated either with enhanced efflux or processing of niclosamide or with apoptosis or autolysis of sporocysts. Of course, more extensive exposure of *B*. *pfeifferi* to niclosamide with attendant loss of the integrity of the snail metabolome would inevitably result in death of *S*. *mansoni* sporocysts as well.

### Shared response of two *Biomphalaria* species to a sublethal dose of niclosamide

Of the 30,647 probe features on the *B*. *glabrata* microarray used by Zhang et al. [16], 16,713 (55%) were homologous to a *B*. *pfeifferi* transcript (Blastn E-value <1e-10, percent identity >75%). Microarray features with homologs to *B*. *pfeifferi* transcripts and that were differentially expressed in both Zhang et al. [16] and the present study are shown in Table 1. These features represent a conservative view of genes characteristic of *Biomphalaria*’s response to sublethal niclosamide exposure. The entire differential expression analysis of *B*. *pfeifferi’*s response to niclosamide showed 895 transcripts up-regulated and 604 down-regulated when compared to uninfected control *B*. *pfeifferi*.

**Table 1:** All snail features shared between *B*. *glabrata* [16] and *B*. *pfeifferi* that were significantly differentially expressed after treated with 0.15mg/L niclosamide.

	B. pfeifferi Illumina transcript	Log_2_FC	B. glabrata array feature	Log_2_FC
ADP-ribosylation factor 3-like	evgTRINITY_DN92963_c1_g2_i1	5.10	c13901	4.73
ADP-ribosylation factor 3-like	evgTRINITY_DN92963_c1_g1_i1	4.45	c13901	4.73
Solute carrier family 28 member 3-like	evgTRINITY_DN88027_c1_g1_i4	6.78	c27272	1.99
Multidrug resistance 1-like	evgTRINITY_BU_DN81217_c7_g4_i1	2.72	contig_14304	1.48
Multidrug resistance 1-like	evgTRINITY_DN90366_c3_g1_i2	5.26	contig_14304	1.48
HSP 12	evglcl|G0WVJSS02FHD9K	2.29	contig_7431	3.79
HSP 12	evglcl|G0WVJSS02JB97J	1.98	contig_7431	3.79
HSP 70	evgTRINITY_GG_25613_c6_g1_i1	1.09	BGC03909	3.64
Solute carrier family 28 member 3-like	evgTRINITY_DN88027_c1_g1_i3	3.79	c27272	1.99
Cytochrome p450	evgTRINITY_BU_DN81631_c8_g1_i1	1.05	c14547_rc	3.10
Cytochrome p450	evgTRINITY_DN93193_c20_g1_i1	2.88	c8814	2.88
Baculoviral IAP repeat-containing 3-like	evgTRINITY_BU_DN78979_c0_g1_i2	1.69	c17676_rc	2.14
Nuclear protein 1-like	evglcl|HJ4YRIA01D0DSV	1.28	contig_4627	2.20
Nuclear protein 1-like	evglcl|HJ4YRIA02HBZUN	1.01	contig_4627	2.20
Growth arrest and DNA damage-inducible alpha-like	evglcl|G0WVJSS02G7JUO	1.85	contig_8438	1.39
Alpha-crystallin B chain	evglcl|HJ4YRIA01ERORD	1.21	contig_2362_rc	1.79
Sequestosome-1-like	evgTRINITY_DN29609_c0_g1_i1	1.00	BGC02302	1.57
Glycogen-binding subunit 76A-like	evgTRINITY_DN70212_c1_g1_i1	0.92	c14016_rc	1.09
Methionine synthase reductase-like	evgTRINITY_DN77579_c0_g1_i1	0.71	c41473	1.00
Glutathione-independent glyoxalase hsp3103	evgTRINITY_DN92822_c15_g1_i1	-1.08	contig_3480	-1.10
Thymidine kinase, cytosolic-like	evgTRINITY_DN90310_c10_g1_i1	-1.29	contig_10981	-1.35
Uncharacterized	evgTRINITY_DN89789_c4_g2_i1	2.39	contig_12514_rc	3.19
Uncharacterized	evglcl|G0WVJSS01A5WAX	3.89	contig_6337_rc	4.16
Uncharacterized	evglcl|G0WVJSS01DEUAY	1.60	contig_3100	2.29
Uncharacterized	evgTRINITY_DN88565_c20_g1_i1	2.00	contig_3944_rc	1.38
Uncharacterized	evgTRINITY_DN22835_c0_g1_i1	1.33	BGC02491	1.02
Uncharacterized	evglcl|G0WVJSS01DKS66	1.36	c43865_rc	1.40
Uncharacterized	evglcl|G0WVJSS02ITT0P	0.82	contig_7634_rc	1.16
Uncharacterized	evgTRINITY_GG_16388_c0_g2_i1	0.86	c13164_rc	1.09
Uncharacterized	evgTRINITY_DN84827_c0_g2_i1	-0.81	c8798_rc	-1.13
Uncharacterized	evgTRINITY_DN93461_c7_g1_i1	-1.49	c1870	-1.80

As a lipophilic xenobiotic, niclosamide would likely be eliminated in animals by increasing its hydrophilicty (phase 1 reaction), conjugating the phase I product with a charged chemical group (phase 2 reaction), and then removing it with the aid of a transmembrane transporter (phase 3 reaction) [47]. A key enzyme superfamily of heme-thiolate proteins responsible for initial phase I detoxification are the cytochrome p450s (CYPs). CYPs are found in all kingdoms of life and most commonly perform monooxygenase reactions adding one oxygen atom to the xenobiotic with the other oxygen atom reduced to water [47]. Zhang et al. [16] found that 9 of the features that were up-regulated ≥ 2-fold change following exposure to 0.15mg/L of niclosamide were CYPs. The *B*. *glabrata* genome has about 99 genes encoding heme-thiolate detoxification enzymes with tissue-specific expression patterns suggesting that CYPs serve specific biological processes [48].

**Fig 2:**
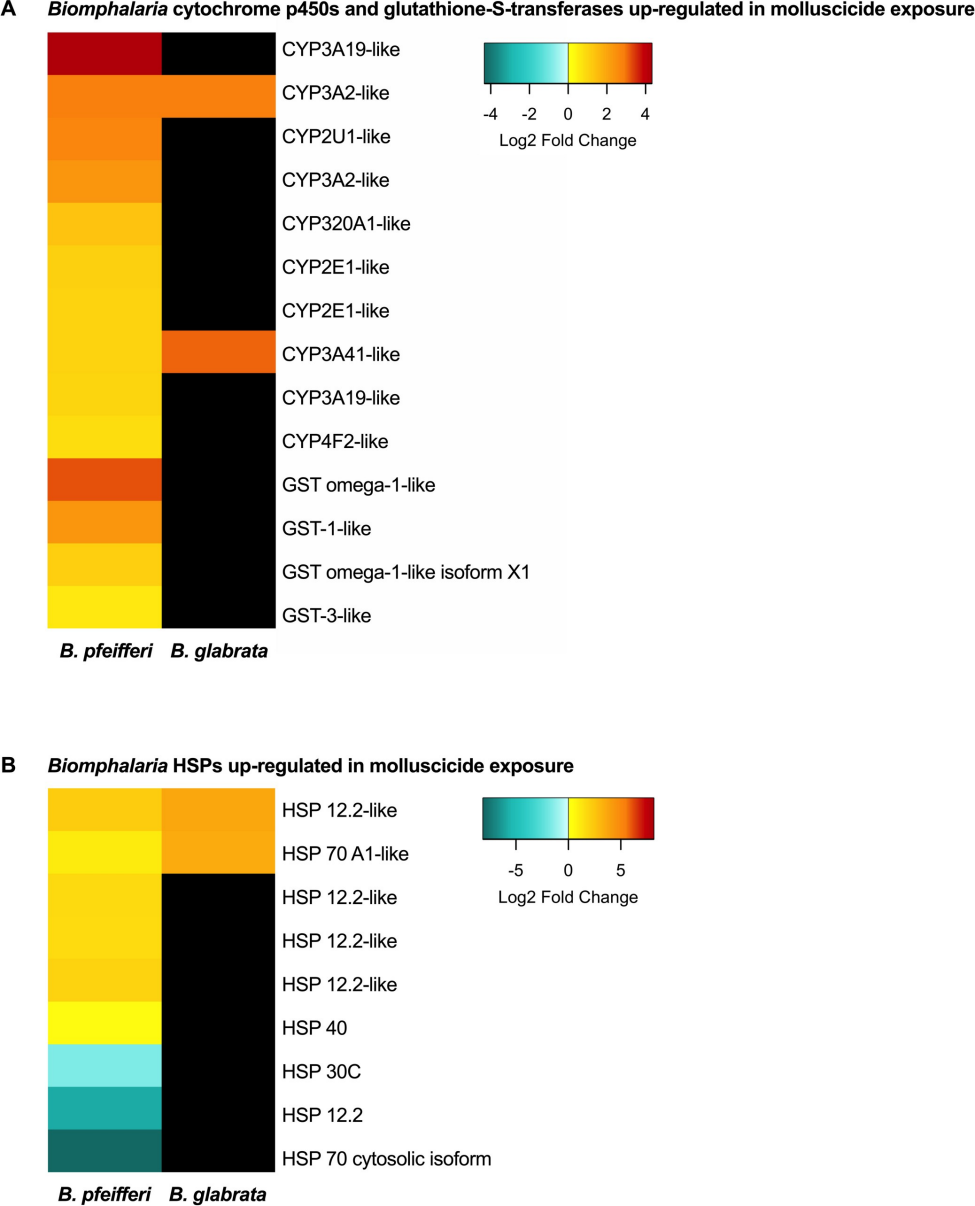
(A) *Biomphalaria pfeifferi* CYP (cytochrome p450s) and GST (glutathione-S-transferases) and (B) *B*. *pfeifferi* heat shock proteins (HSPs) up-regulated in response to sublethal niclosamide treatment. Data for *B*. *glabrata* from Zhang et al. [16].

CYPs are also up-regulated in *B*. *pfeifferi* in response to niclosamide exposure, including two in common with *B*. *glabrata* (Table 1) and 8 more as noted in Fig 2A, underscoring the importance of CYP mixed function oxidases in the snail response to niclosamide. Of the CYPs up-regulated in both snail species, one is a homolog of Cp450 3A2-like found in mouse liver cell microsomes which is responsible for oxidizing steroids, fatty acids, and xenobiotics. The other shared CYP is CYP 3A41-like. It is also microsomal and studies of vertebrate homologs indicate that glucocorticoids may exert control of CYP3A41 gene expression [49]. Modest down-regulation of one CYP in *B*. *glabrata* (CYP II f2) was also observed [16] and we similarly noted down-regulation of a CYP (1-like isoform X1) in *B*. *pfeifferi*. This supports the suggestion by Zhang et al. [16] that different members of the CYPs repertoire are likely to have different functions in *Biomphalaria* snails in response to diverse stimuli, including biotic challenges like *S*. *mansoni* or abiotic challenges like molluscicides.

Phase 2 in the elimination of xenobiotics would likely involve molecules like glutathione transferases that transfer charged chemical species like glutathione to the xenobiotic. Glutathione-S-transferase 7-like (GST) was up-regulated 5-fold following niclosamide exposure in *B*. *glabrata* [16]. In addition to CYPs, GST has also been shown to be up-regulated following niclosamide-based molluscicide exposure in *Oncomelania hupensis* [15]. GST was also represented in the *B*. *pfeifferi* Illumina DE transcripts with up-regulation of GST omega-1-like, and microsomal GST-1 and -3-like (Fig 2A).

Transmembrane transporters complement the detoxification and conjugation reactions of phases 1 and 2 by eliminating the xenobiotic or toxin present in an organism [47]. ATP-binding cassette (ABC) transporters, particularly ABC efflux transporters, play an important role in eliminating toxic compounds from cells. For instance, ABCG2, a non-specific, multi-xenobiotic transporter is known to be expressed at high levels in the gills and hemocytes of *Mytilus edulis* [50]. One family of ABC efflux transporters, the multidrug resistance proteins (MRPs) act to eliminate drugs and toxic chemicals transporting anionic compounds detoxified in phases 1 and 2. One MRP-1 is expressed 2.8-fold higher than controls in *B*. *glabrata* [16] and 10 MRP-1 transcripts were up-regulated in *B*. *pfeifferi* suggesting these transporters are removing toxic waste products produced directly by niclosamide or indirectly through cell death or tissue necrosis (S2 Table).

Heat shock proteins are up-regulated after exposure to a variety of stressors including elevated temperature, hypoxia, ischemia, heavy metals, radiation, calcium increase, glucose deprivation, various pollutants, drugs, and infections [51]. Up-regulation of HSPs has been associated with susceptibility of *B*. *glabrata* to *S*. *mansoni* [52–53]. HSPs have also been identified in other molluscs as indicators of environmental stress. The disk abalone *Haliotis discus discus* up-regulates HSP 20 when treated with extreme temperatures, changing salinity, heavy metals, and microbial infection [54]. The marine bivalve, *Mytilus galloprovincialis* up-regulates HSPs 24.1, 70, 90, and sequestosome-1 following toxic metal exposure [55]. *Biomphalaria glabrata* mounts a multifaceted HSP response to niclosamide by up-regulating HSPs 12, 40, and 70 [16] and the selective autophagosome cargo protein sequestosome-1. We also saw up-regulation of these specific HSPs but the more comprehensive sequencing available from the Illumina study revealed mixed responses of isoforms of HSP 12.2 and down-regulation of HSP 30 (Fig 2B).

In response to exposure to *S*. *mansoni* infection, *B*. *pfeifferi* shows a more complex transcriptional expression of HSPs, cytochrome p450s, and glutathione-S-transferases than it does to molluscicide with no general up- or down-regulation of any group of these transcripts [23]. Biotic stressors such as parasites with intimate and prolonged contact with host tissues may induce a more complex stress response with up- and down-regulation of various HSPs in comparison to a general up-regulation of CYPs, glutathione-S-transferases, small and large molecular weight HSPs, and sequestosome noted in the response of several molluscs to abiotic stressors.

### Additional responses of *Biomphalaria* to sublethal molluscicide exposure detected with Illumina RNA-Seq

Transcripts involved in protection from oxidative damage, generalized pathogen defense and innate immunity, protease inhibitors and feeding behavior were all noted. We observed high expression of several glutathione peroxidase transcripts, presumably associated with enhanced conversion of hydrogen peroxide to water. In cancerous colon cells, niclosamide increased cell death when used with a therapeutic drug through hydrogen peroxide production [56], therefore, it is not inconceivable that niclosamide in snails is directly or indirectly involved in increasing hydrogen peroxide levels. Glutathione peroxidase has been shown to increase the general tolerance of cells to oxidative stress resulting from exposure to xenobiotics [57].

Glutathione reductase, a critical oxidoreductase enzyme that catalyzes the reduction of glutathione disulfide to glutathione, surprisingly was down-regulated. As noted above, glutathione is a key ingredient needed in phase II conjugation mediated by the enzyme glutathione-S-transferase, which is up-regulated in *B*. *pfeifferi* following molluscicide exposure. An impaired ability to regenerate glutathione because of down-regulated glutathione reductase activity could then impair both the detoxification process and interfere with maintenance of redox balance by allowing hydrogen peroxide to accumulate.

One of the more striking responses of *B*. *pfeifferi* treated with niclosamide was the high up-regulation of transcripts for several protease inhibitors including antitrypsin-like and serpins (serine protease inhibitors) and the down-regulation of metallo, cysteine, and serine proteases. In contrast, only one serine protease (chymotrypsin-like elastase family member 1) and a single aminopeptidase N-like transcript were up-regulated. Caspases are cysteine-dependent proteases that play essential roles in programmed cell death [58] and isoforms of caspase-2 and 3 were down-regulated in niclosamide-exposed *B*. *pfeifferi*. The down-regulation of protease activity may be part of a compensatory stress response made by the snail to minimize metabolic changes associated with niclosamide exposure that if left unchecked would lead to apoptosis and protein degradation.

Responses typically classified as innate immune responses because they occur following exposure to parasites like *S*. *ma*nsoni were also noted in *B*. *pfeifferi* exposed only to niclosamide. One such transcript was homologous to CD109 antigen-like, a thioester-containing protein, which is highly enriched in plasma from both resistant and susceptible strains of *B*. *glabrata* containing miracidia transforming into mother sporocysts [59]. We also noted up-regulation of a transcript identified as complement C1q-like protein that we have reported to be up-regulated in early *S*. *mansoni-*infected *B*. *pfeifferi* [23]. Fibrinogen-related proteins (FREPs) 1 and 2 were both up-regulated after niclosamide exposure; FREP2 was also up-regulated in *S*. *mansoni*-shedding *B*. *pfeifferi* [23]. Dermatopontin, a parasite-responsive gene frequently noted in studies of both *B*. *glabrata* and *B*. *pfeifferi*, was also up-regulated following niclosamide exposure.

A conspicuous response was the high up-regulation of over 100 diverse transcripts identified as LBP/BPI1 (lipopolysaccharide binding protein/bacterial permeability-increasing protein 1) in *B*. *pfeifferi* after exposure to niclosamide. LBP/BPI1 is an antimicrobial molecule found in the albumen gland of *B*. *glabrata* and egg masses [60]. Silencing of LBP/BPI1 expression in *B*. *glabrata* resulted in significant reduction of egg-laying, and death of eggs attributable to oomycete infections, providing evidence that LBP/BPI is involved in parental immune protection of offspring [61].

Transcripts homologous to *B*. *glabrata* tyrosinases (Tyr) 1, 2, and 3, are also up-regulated in response to niclosamide. In early-stage pre-patent *S*. *mansoni* infections Tyr-1 is up-regulated, and Tyr-3 is down-regulated in *B*. *pfeifferi* harboring cercariae-producing sporocysts [24]. Tyrosinases are involved in melanin synthesis and additionally might mark an early phase in initiation of castration by diverting tyrosine towards the production of melanin instead of dopamine in *S*. *mansoni*-infected *B*. *pfeifferi* [23]. Like LBP/BPI1, tyrosinase has also been isolated from *B*. *glabrata* egg masses and is presumed to provide an immunoprotective effect for developing embryos by contributing to the melanization of the egg membrane [60,62]. The additional considerable effort by the snail to make two egg mass-associated proteins in response to niclosamide is baffling, but might represent a last-ditch attempt to produce offspring before death. Alternatively, perhaps this is best viewed as an example of relatively non-specific innate immune responses that can be invoked by exposure to an unusual stressor, even if it is of an abiotic nature. Another consideration is that it represents a response to the presence of bacteria in the snail that might appear due to impaired hemocyte function or possibly due to failure to contain the gut microbiome in its usual compartment.

Another unexpected response was the high up-regulation of myomodulin-like neuropeptide in niclosamide-treated *B*. *pfeifferi*. Myomodulins are neurotransmitters involved in regulating feeding behavior by controlling radula protractor muscles used for feeding [63] in *Lymnaea stagnalis* [63,64] and *Aplysia californica* [65]. Myomodulin is down-regulated in pre-patent *S*. *mansoni*-infected *B*. *glabrata* and this was implicated as possibly diminishing feeding efficiency in infected snails [66]. Down-regulation of a *B*. *pfeifferi* feeding circuitry peptide was seen in early and patent *S*. *mansoni* infections [23]. The up-regulated myomodulin activity noted here provides evidence that basic physiological activities such as feeding are altered after niclosamide exposure. The mussel *Mytilus edulis* shows a decreased rate of feeding after exposure to hydrophobic organic chemicals, organochlorine compounds, organophosphate and carbamate pesticides, and pyrethroids [67–68].

With respect to features down-regulated following niclosamide exposure, it would seem transcription and translation efficiency would be hindered as evidenced by down-regulation of nearly a dozen ribosomal proteins, transcription factors, and mitogen-activated protein kinases (MAPKs). Of transcripts associated with stress responses, HSP 30 and HSP 70 cytosolic isoform were down-regulated along with an HSP 12 isoform. Neuroglobins are members of the hemoglobin superfamily of oxygen carriers, are expressed in the glial cells surrounding neurons and have been found in marine, freshwater, and terrestrial molluscs including the gastropods *L*. *stagnalis*, *Planorbis corneus*, *A*. *californica*, *Helix pomatia* and *Cepaea nemoralis* [69]. Although we did not observe down-regulation of the hemoglobin-encoding gene noted by Zhang et al. [16] following exposure of *B*. *glabrata* to niclosamide, down-regulation of neuroglobin in niclosamide-exposed *B*. *pfeifferi* was observed. This could be associated with reduced availability of oxygen, at least for neural cells.

Significant down-regulation of a Cu-Zn SOD (-9.3 log_2_FC) in *B*. *pfeifferi* indicates that SODs have a more complex response to niclosamide than previously thought from the microarray study by Zhang et al. [16]. High expression of certain alleles of Cu-Zn SOD have been implicated in resistance of *B*. *glabrata* strain 13-16-R1 to *S*. *mansoni* [70–72] so it is not unlikely that different Cu-Zn SODs show distinctive responses to other stressors like niclosamide. Calmodulins, ubiquitous calcium-dependent signaling proteins responsible for regulating the uptake, transport, and secretion of calcium in gastropod shell formation [73–74], are expressed by *B*. *glabrata* in response to gram (-) and gram (+) bacteria, yeast [75], and in *B*. *glabrata* snail plasma containing larval *S*. *mansoni*. Here, we saw down-regulation of calmodulin in *B*. *pfeifferi* treated with the niclosamide, raising the possibility that calmodulin expression is more responsive to biotic challenges. Transcripts related to cell adhesion like spondins that are expressed in *Biomphalaria* hemocytes [76] were also down-regulated.

### Responses of *B*. *pfeifferi* with cercariae-producing *S*. *mansoni* infections to sublethal niclosamide treatment

As previously noted, snails treated with the combined effects of the biological stressor *S*. *mansoni* and the abiotic stressor niclosamide were surprisingly responsive (Fig 1), exhibiting large numbers of uniquely up- and down-regulated features, with many of these only modest in the degree of their differential expression. Among the more notable responses were several features associated with managing cell death in damaged tissues (Table 2). The transmembrane transporter ABCA3 is associated with resistance to xenobiotics and engulfment during apoptosis [77]. The enzymes glutaredoxin-2-like and catalase-like are both involved in reduction of hydrogen peroxide that may be released during niclosamide-induced apoptosis. An increase in apoptosis could account for the up-regulation of lysosomal endopeptidases such as cathepsin-L-like. Two mitochondria-associated transcripts that also play a role in gluconeogenesis, glyceraldehyde-3-phosphate dehydrogenase (GAPDH) and glycerol-3-phosphate dehydrogenase-like (GPDH) were also up-regulated. GAPDH accumulates in mitochondria during apoptosis and induces pro-apoptotic mitochondrial membrane permeability [78]. Niclosamide has been screened as a potential promoter of mitochondrial fragmentation by disrupting membrane potential, reducing ATP levels, and inducing apoptosis by caspase-3-activation in HeLa cells [79].

**Table 2:** *Biomphalaria pfeifferi* transcripts up-regulated in response to dual stressors (*S*. *mansoni* infection and sublethal niclosamide exposure) identified for their potential role in programmed cell death. Except where noted, functions were obtained from Entrez Gene at https://www.ncbi.nlm.nih.gov/gene and UniProtKB at www.uniprot.org/uniprot.

Transcript Description	Function
ABCA3 transmembrane transporter	Resistance to xenobiotics and engulfment during apoptosis
Growth arrest-specific protein 2-like	Cell cycle arrest; regulation of cell shape; may act as a cell death substrate for caspases
Glutaredoxin-2-like	Mitochondrial; response to hydrogen peroxide and regulation of apoptosis caused by oxidative stress
Calmodulin 2/4-like, 5, A-like	Can mediate the stress response calcium-dependent signaling that controls a variety of enzymes, ion channels, proteins, kinases, and phosphatases
Heparanase-like	Facilitates cell migration associated with metastasis, wound healing and inflammation
Catalase-like	Reduction of hydrogen peroxide
Caspase 3 and 8-like	TNF binding; endopeptidase activity involved in apoptosis
Tumor necrosis factor (TNF) and receptor	Induces cell death
Cathepsin-L-like	Lysosomal endopeptidase
Glyceraldehyde-3-phosphate dehydrogenase (GAPDH)	Induces pro-apoptotic mitochondrial membrane permeability (Deniaud et al. 2007)

Pattern recognition receptors (PRRs), key elements responsible for the recognition of pathogens, showed mixed responses. Four distinct PRR genes were up-regulated: peptidoglycan-recognition protein SC2-like, ficolin-like, FREP 2, and FREP 10. We have reported the up-regulation of FREP 2 in *S*. *mansoni*-infected *B*. *pfeifferi* [24] but here we see four additional isoforms of FREP 2 up-regulated. Toll-like receptors (TLRs) which are involved in recognizing pathogens and activating conserved innate immune signaling pathways [80], were conspicuously down-regulated (TLRs 3, 4, 5, 7, and 8). Additional transcripts that function in various aspects of innate immune responses and that were down-regulated are C3 PZP-like alpha-2-macroglobulin domain-containing protein 8, hemolymph trypsin inhibitor B-like, tyrosine-3-monooxygenase, DBH-like monooxygenase 2, and tyramine beta-hydroxylase-like.

As with snails treated with niclosamide alone, once again a down-regulation of transcripts for ribosomal proteins was noted. Reduction in ribosome production can be considered a stress response because it is a rapid and effective response against misfolded proteins [81] but may simply be an indication of a downgrading of general condition. Other down-regulated transcripts show diverse functional activity. Several annexins, intracellular Ca^2+^ and phospholipid binding proteins are down-regulated showing the possible disruption of regulation of membrane organization, trafficking, and the regulation of Ca^2+^ concentrations within cells [82].

Unlike the general up-regulation of CYPs in *B*. *pfeifferi* exposed only to niclosamide, *B*. *pfeifferi* with dual stressors highly down-regulate several CYPs (microsomal CYPs 2J1-like, 2B4-like, 3A29-like, 26A1-like, and mitochondrial CYP12A2-like). Mitochondrial CYP12A2-like is known to metabolize a variety of insecticides and xenobiotics [83]. We cannot discount that contribution to the down-regulation of this particular CYP is a result of mitochondrial degradation caused, in part, by niclosamide as noted previously as well as the additional stress of a patent *S*. *mansoni* infection.

### Concluding remarks

This study provides a distinctive and detailed view of the nature of the response of field-derived *B*. *pfeifferi* to relevant stressors likely to be encountered in its environments, including infections with *S*. *mansoni*, just one of several digenetic trematodes known to commonly infect this snail in Africa [84], and treatment with the commonly used molluscicide, niclosamide. It is important to gain additional detailed information regarding the effects of niclosamide on snails, particularly those that harbor schistosome infections. For example, do infected snails succumb more readily to treatment and if so, why? This particular aspect of molluscicide use has not been widely investigated.

In general, treatment with niclosamide alone resulted in the fewest responsive features in *B*. *pfeifferi* (1,711) followed by infection with *S*. *mansoni* (2,271) and then by the combination of niclosamide and *S*. *mansoni* (7,683). Snails in these three groups all responded in very distinct ways, but in each case with more features up- than down-regulated. Sublethal exposure to a single xenobiotic provoked about 67% as large a transcriptomic response as was noted for snails shedding *S*. *mansoni* cercariae, snails that had probably been infected with the parasite for at least a month and harbored large numbers of daughter sporocysts. The fact that snails that received the combination of infection and niclosamide responded so much more vigorously with so many distinctive features suggests that they were under greater duress and that their responses in some sense preempted the responses of snails in the other two groups.

Treatment with niclosamide alone provoked up-regulation of several features associated with response to xenobiotics including cytochrome p450s, heat shock proteins, multidrug resistant transporters and glutathione-S-transferases, confirming many of the observations made by Zhang et al [16] in a microarray study of *B*. *glabrata* treated with sublethal doses of niclosamide. Several additional unique aspects of the response to niclosamide were also noted given the increased resolution provided by Illumina sequencing. We note that one of the effects of niclosamide on *B*. *pfeifferi* may be to contribute to redox imbalance because glutathione is being used by glutathione-S-transferases to conjugate xenobiotics but may not be sufficiently regenerated because of down-regulated activity of glutathione reductase.

Exposure of infected snails to niclosamide was noteworthy in revealing the involvement of several features not found to be responsive to either stressor alone. Although many of the uniquely expressed features did not respond dramatically, the ones that did were indicative of responses associated with apoptosis, reduced protein synthesis, reduced production of some CYPs and thus diminished detoxification ability, and diminished innate immune function. Accordingly, we hypothesize that the combination of stressors was likely overcoming the snail’s ability to maintain homeostasis. The snail mounts a considerable transcriptomic response to the presence of cercariae-producing sporocysts [23] and it is not hard to imagine that the energy demand placed on infected snails by continual production of cercariae takes an additional toll. The mortality rate of *B*. *pfeifferi* infected with *S*. *mansoni* is significantly higher than that noted for unexposed control snails [25]. The molluscicide-exposed infected snails selected for sequencing were alive when sampled, but the transcriptional profiles suggested they were not thriving. This is broadly in agreement with observations made to indicate that *B*. *sudanica* with *S*. *mansoni* infections succumb to sublethal niclosamide treatment at a higher rate than do uninfected controls [20]. In other words, the combination of stressors used here exposed the limits of what these snails can do to maintain homeostasis.

We remind readers of three possible scenarios presented in the introduction regarding *S*. *mansoni* transcriptional response to molluscicide: 1) An overall absence of *S*. *mansoni* transcripts indicating suspension of activity; 2) Cercariae-producing *S*. *mansoni* sporocysts express unique features that are absent in response to molluscicide exposure; and 3) Shedding *S*. *mansoni* stages treated with molluscicide show transcriptional responses suggestive of an ability to protect the host-parasite unit in which they reside from a xenobiotic. We can discount the first scenario because the sporocyst response did not appear to be as indicative of a failure to maintain homeostasis as we noted for snails. This is in keeping with the general observation that the lethal dose of niclosamide for sporocysts is probably higher than for snails [20]. Although it is clear that both miracidia and cercariae are vulnerable to niclosamide [17–18], this may be a reflection of their more aerobic metabolism and that they would be more fully exposed to the action of niclosamide *in vitro* as compared to sporocysts nested within the tissues of an infected snail. Inspection of the transcripts produced uniquely by niclosamide-exposed sporocysts does not reveal any candidates that would seem to favor resilience to niclosamide. This coupled with the stable expression of known defense or stress response genes noted above leads us to a conclusion that sporocysts have little if any ability to mount protective responses to niclosamide and certainly do not seem to provide anything that would favor enhanced survival of their host snail in the presence of a chemical that is clearly lethal for the host. It is possible that the parasite can only rely on host xenobiotic detoxification capabilities when confronted with niclosamide. Regarding scenario #3, even though *S*. *mansoni* sporocysts within snails treated with niclosamide expressed more transcripts than in untreated snails, there was little about the response to suggest they possessed any distinctive or large-scale ability to respond to a xenobiotic like niclosamide, so it can be rejected. We can though accept a modified version of our second scenario as our data suggests niclosamide treatment to *B*. *pfeifferi* with cercariae-producing *S*. *mansoni* sporocysts does not produce a strong negative effect on the transcriptomic responses of sporocysts. However, given the relatively unhealthy state of the treated snails, it would inevitably follow that the condition of the sporocysts would degenerate.

The broader implications of the current and future use of molluscicides for snail control remain unknown; however, the threat remains that snails downstream from the point of treatment could survive being exposed to lower doses (a result of dilution) of molluscicide, thus remaining susceptible to infection [20]. It remains to be seen if individual snails that happen to be repeatedly exposed to sublethal doses of niclosamide might experience faster and more durable induction of protective compounds, rendering them more resistant to later lethal doses. Also, by either enhancing or normal immune function of the snail, sublethal molluscicide treatment could potentially alter the normal balance of the snail-schistosome interaction, possibly increasing or diminishing compatibility. Or, in snails with pre-existing *S*. *mansoni* infections, sporocysts may capitalize on altered snail defenses resulting from molluscicide treatment and potentially increases cercarial production. Possibilities like these should be taken into consideration in the planning of snail control programs that use molluscicides such as niclosamide in situations where their concentrations are rapidly diluted in large water volumes, or where molluscicicdes are repeatedly applied. Further study is necessary to determine if these are realistic possibilities.

In conclusion, we noted remarkably distinctive transcriptomics responses for *B*. *pfeifferi* depending on the nature of the stressor they received, and that the combination of niclosamide and *S*. *mansoni* infection imposed a level of stress on the snails that resulted in an extensive response comprised of many features we had not observed previously. This study contributes to the growing list of molecular participants that may govern the outcomes of the intimate interrelationships between snails and schistosomes, and that may help us understand how snail host biology might be targeted for disruption by molluscicidal chemicals.

## Supporting information

S1 File Significantly (PPDE > = 0.95) up- and down-regulated *B*. *pfeifferi* transcripts used in the analyses. (XLSX)

S2 File *Schistosoma mansoni* transcripts expressed in cercariae-producing sporocysts in *B*. *pfeifferi* treated with sub-lethal molluscicide. (XLSX)

S1 Table Details of field-collected samples used in this study including abbreviation used in supplementary files 1 and 2 (S1 File, S2 File), designation of samples that had a sublethal molluscicide treatment, the number of replicates in each group, and the number of paired-end reads recovered post-quality filtering (as described in [23]). (DOCX)

S2 Table *Biomphalaria pfeifferi* multidrug resistant protein 1-like transcripts up-regulated after treatment with sublethal niclosamide. (DOCX)

S1 Fig Principle component plot (PCA) for the two groups compared in our analyses: *B*. *pfeifferi* shedding *S*. *mansoni* untreated (Shedding) and *B*. *pfeifferi* shedding *S*. *mansoni* treated with a sub-lethal molluscicide dose (ShedMoll). (TIF)

S2 Fig (A) *Schistosoma mansoni* transcripts expressed per replicate in snails shedding *S. mansoni* untreated (Shedding) and snails shedding *S. mansoni* and treated with molluscicide. (B) Venn diagram of shared and unique *S. mansoni* transcripts in treated and untreated groups. (C) Frequency distribution of log2-transformed TPMs (transcripts per million) of *S. mansoni*.
(TIF)

S3 Fig Stress and defense transcripts expressed by *S*. *mansoni* untreated (Shedding) and molluscicide-treated (Shedding + Molluscicide) including homologs of planarian bacterial defense factors, heat shock proteins, peroxiredoxins, SODs (superoxide dismutases), and cytochrome p450. (TIF)

S4 Fig ABC transporters expressed by *S*. *mansoni* in *S*. *mansoni*-shedding samples (Shedding) and *S*. *mansoni*-shedding samples treated with a sublethal dose of niclosamide (0.15mg/L) (Shedding + Molluscicide). Expression is measured as log_2_-transformed TPM (transcripts per million) and ordered by hierarchical clustering.
(TIF)

S5 Fig *Schistosoma mansoni* proteases and protease inhibitor transcripts expressed in *S*. *mansoni*-shedding samples (Shedding) and *S*. *mansoni*-shedding samples treated with a sublethal dose of niclosamide (0.15mg/L) (Shedding + Molluscicide). (TIF)

S6 Fig Intramolluscan *S. mansoni* of *B. pfeifferi* treated with molluscicide (Shedding + Molluscicide) exhibited modest increases in expression of cercarial elastases (SmCE1a, SmCE1a.2, cercarial protease, and SmCE2b) (A), nutrient transporters (glucose, amino acid, and nucleoside) (B), germinal cell proliferation (C), and neural development and neuropeptides (D). Shedding *S. mansoni* stages treated with niclosamide had higher transcript levels for cell polarity protein, neuronal differentiation, notch, SOX transcription factor, and septate junction protein and although modest, these may have important downstream effects on germinal cell proliferation or neurogenesis.
(TIF)
