# Supplementary material for: Transcriptional responses of Biomphalaria pfeifferi and Schistosoma mansoni following exposure to niclosamide, with evidence for a synergistic effect on snails following exposure to both stressors
Source: PLoS Negl Trop Dis. 2019 Dec 16;13(12):e0006927. doi: 10.1371/journal.pntd.0006927 (PMC6936870; doi:10.1371/journal.pntd.0006927)
Supplement: S1 Table — (DOCX) [file pntd.0006927.s003.docx]

| Field-collected samples | Abbreviation | Molluscicide | Replicate | Paired-end reads  (post-quality filtering) |
| --- | --- | --- | --- | --- |
| *B. pfeifferi* uninfected |  |  | 1 | 28,903,992 |
|  | Bp | Untreated | 2 | 34,318,971 |
|  |  |  | 3 | 27,557,936 |
| *B. pfeifferi* shedding *S. mansoni* |  |  | 1 | 32,200,842 |
|  | BpSm | Untreated | 2 | 33,570,583 |
|  |  |  | 3 | 27,569,638 |
| *B. pfeifferi* uninfected x molluscicide |  |  | 1 | 35,289,769 |
|  | BpMoll | Treated | 2 | 34,450,509 |
|  |  |  | 3 | 25,652,418 |
| *B. pfeifferi* shedding  *S. mansoni* x molluscicide |  |  | 1 | 30,587,208 |
|  | BpSmMoll | Treated | 2 | 35,071,339 |
|  |  |  | 3 | 28,843,961 |

S1 Table.
