# Supplementary material for: Transcriptional responses of Biomphalaria pfeifferi and Schistosoma mansoni following exposure to niclosamide, with evidence for a synergistic effect on snails following exposure to both stressors
Source: PLoS Negl Trop Dis. 2019 Dec 16;13(12):e0006927. doi: 10.1371/journal.pntd.0006927 (PMC6936870; doi:10.1371/journal.pntd.0006927)
Supplement: S2 Table — (DOCX) [file pntd.0006927.s004.docx]

S2 Table.

| Up-regulated *B. pfeifferi* MRP transcripts | Log_2_ FC |
| --- | --- |
| evgTRINITY_GG_18090_c5_g2_i1 | 7.8 |
| evgTRINITY_BU_DN63065_c0_g1_i1 | 5.7 |
| evgTRINITY_BU_DN81217_c7_g4_i3 | 5.7 |
| evgTRINITY_DN90366_c3_g1_i2 | 5.3 |
| evgTRINITY_DN90366_c3_g1_i1 | 5.0 |
| evgTRINITY_BU_DN81217_c7_g4_i1 | 2.7 |
| evgTRINITY_DN1870_c0_g1_i1 | 2.2 |
| evgTRINITY_DN92909_c10_g1_i1 | 1.4 |
| evgTRINITY_DN87672_c0_g1_i1 | 1.3 |
| evgTRINITY_DN84897_c0_g1_i1 | 1.3 |
