# Supplementary figures and images for: Transcriptional responses of Biomphalaria pfeifferi and Schistosoma mansoni following exposure to niclosamide, with evidence for a synergistic effect on snails following exposure to both stressors
Source: PLoS Negl Trop Dis. 2019 Dec 16;13(12):e0006927. doi: 10.1371/journal.pntd.0006927 (PMC6936870; doi:10.1371/journal.pntd.0006927)

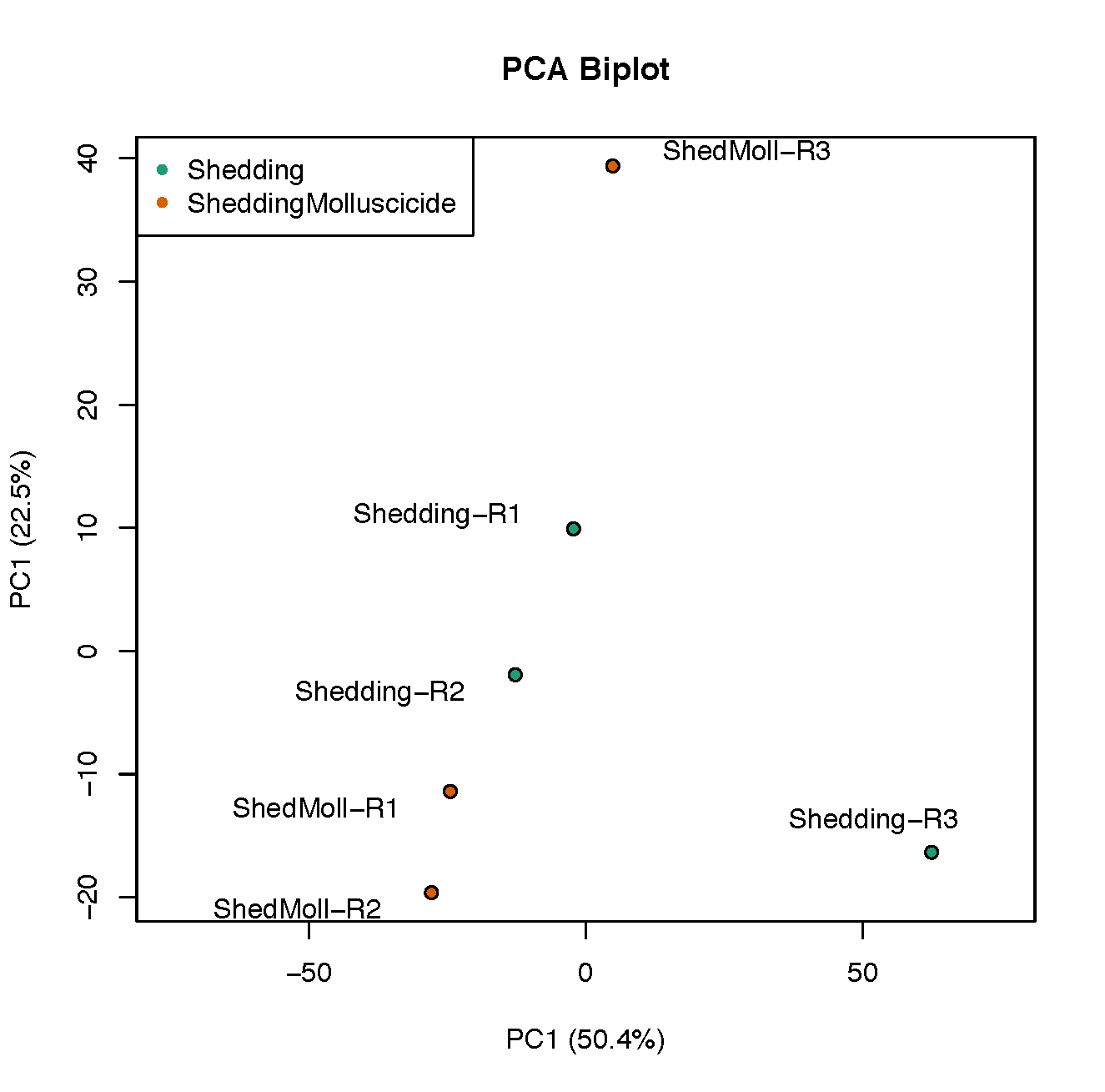

Supplement: S1 Fig — (TIF) [file pntd.0006927.s005.tif]

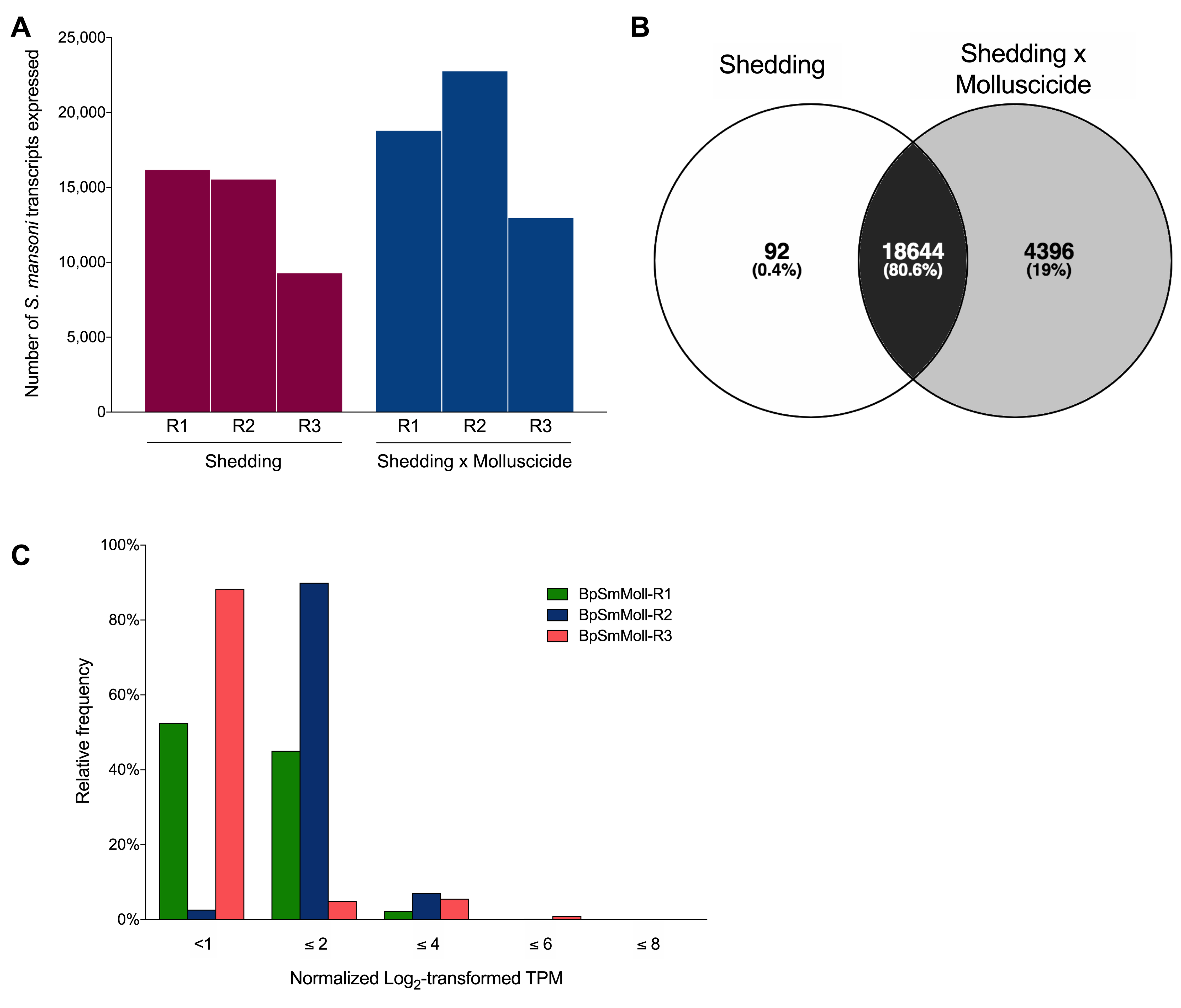

Supplement: S2 Fig — (A) Schistosoma mansoni transcripts expressed per replicate in snails shedding S. mansoni untreated (Shedding) and snails shedding S. mansoni and treated with molluscicide. (B) Venn diagram of shared and unique S. mansoni transcripts in treated and untreated groups. (C) Frequency distribution of log2-transformed TPMs (transcripts per million) of S. mansoni. (TIF) [file pntd.0006927.s006.tif]

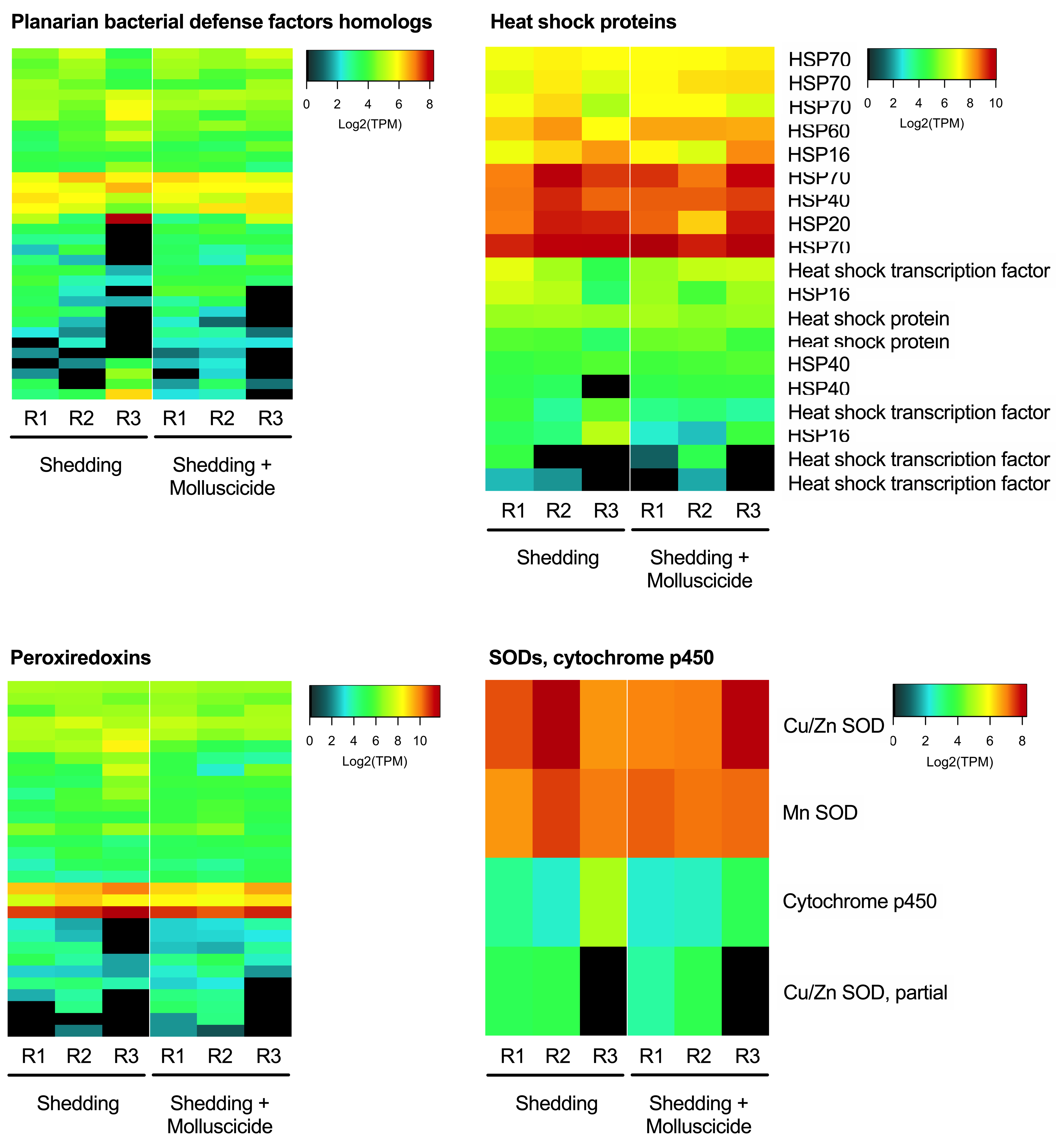

Supplement: S3 Fig — (TIF) [file pntd.0006927.s007.tif]

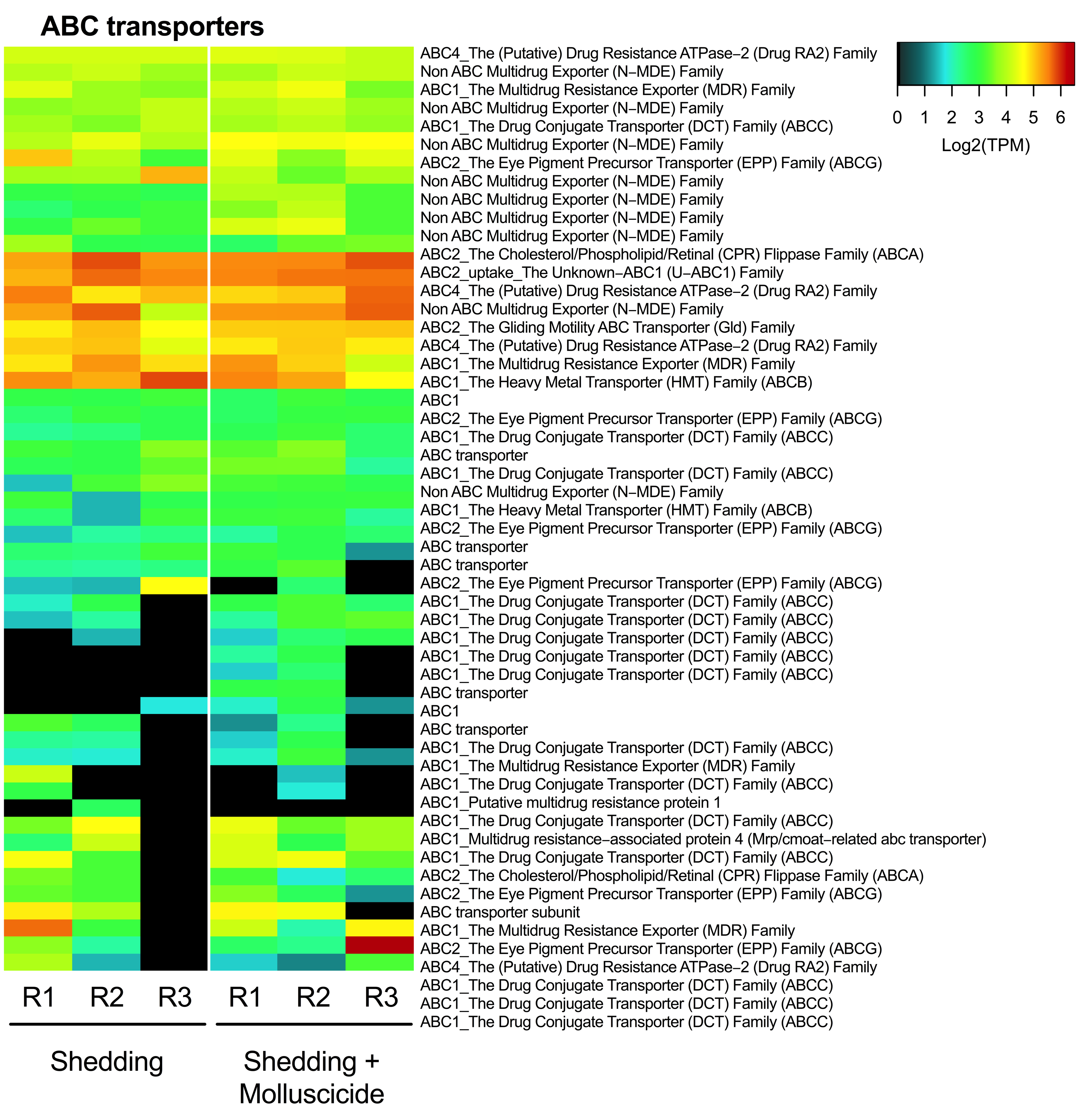

Supplement: S4 Fig — Expression is measured as log2-transformed TPM (transcripts per million) and ordered by hierarchical clustering. (TIF) [file pntd.0006927.s008.tif]

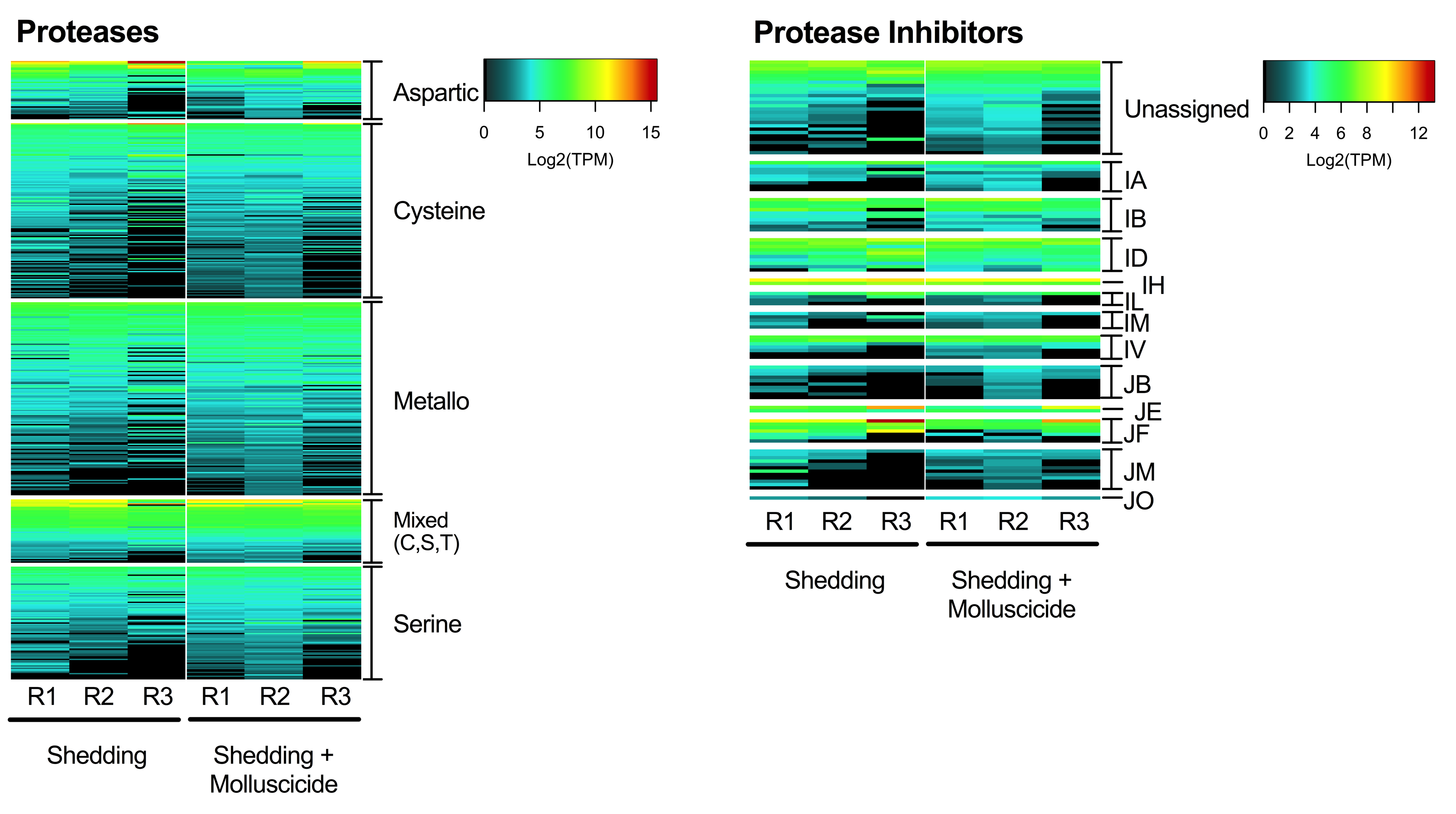

Supplement: S5 Fig — (TIF) [file pntd.0006927.s009.tif]

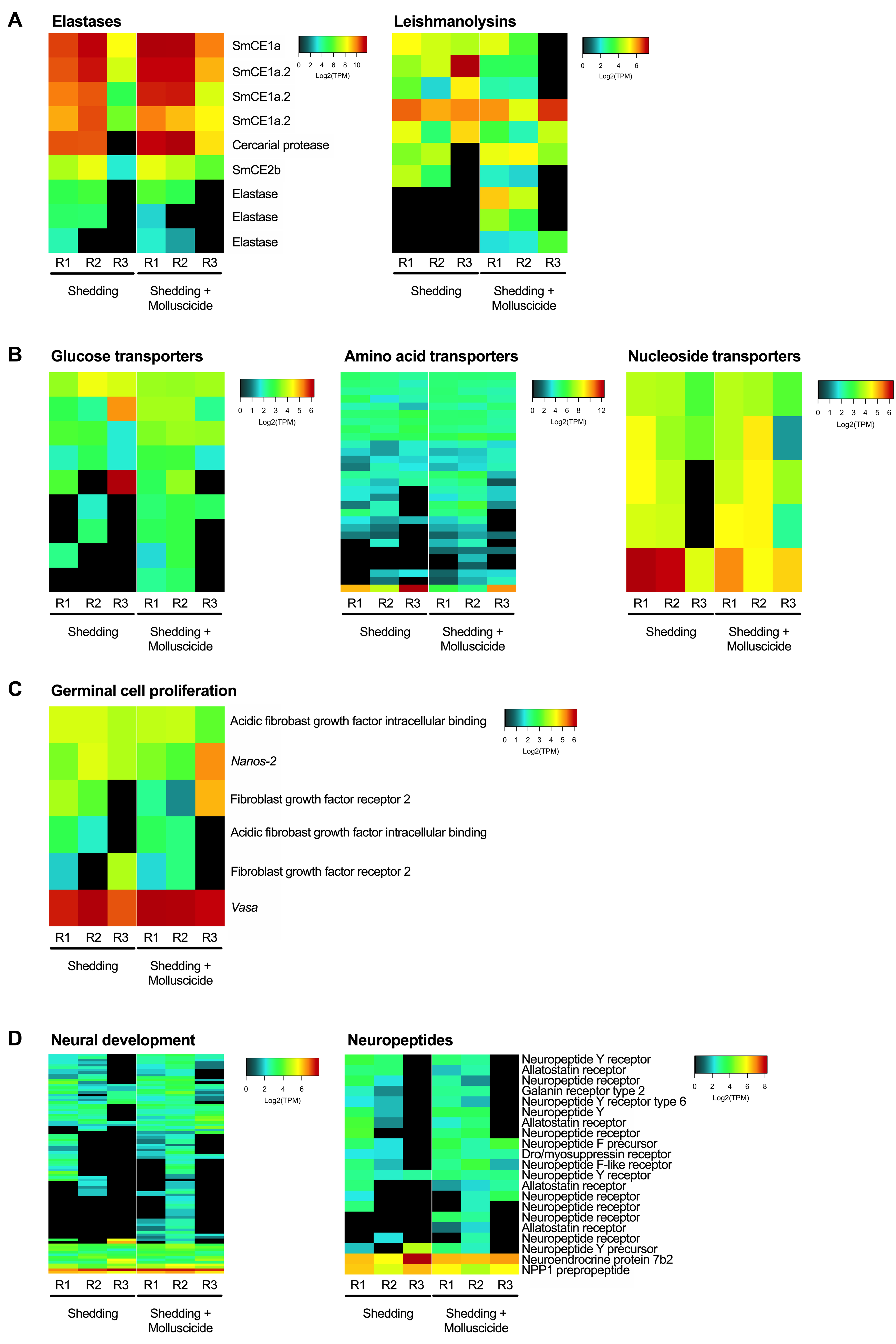

Supplement: S6 Fig — Intramolluscan S. mansoni of B. pfeifferi treated with molluscicide (Shedding + Molluscicide) exhibited modest increases in expression of cercarial elastases (SmCE1a, SmCE1a.2, cercarial protease, and SmCE2b) (A), nutrient transporters (glucose, amino acid, and nucleoside) (B), germinal cell proliferation (C), and neural development and neuropeptides (D). Shedding S. mansoni stages treated with niclosamide had higher transcript levels for cell polarity protein, neuronal differentiation, notch, SOX transcription factor, and septate junction protein and although modest, these may have important downstream effects on germinal cell proliferation or neurogenesis. (TIF) [file pntd.0006927.s010.tif]
